# Kirsten ras mutations in patients with colorectal cancer: the ‘RASCAL II’ study

**DOI:** 10.1054/bjoc.2001.1964

**Published:** 2001-09

**Authors:** H J N Andreyev, A R Norman, D Cunningham, J Oates, B R Dix, B J Iacopetta, J Young, T Walsh, R Ward, N Hawkins, M Beranek, P Jandik, R Benamouzig, E Jullian, P Laurent-Puig, S Olschwang, O Muller, I Hoffmann, H M Rabes, C Zietz, C Troungos, C Valavanis, S T Yuen, J W C Ho, C T Croke, D P O'Donoghue, W Giaretti, A Rapallo, A Russo, V Bazan, M Tanaka, K Omura, T Azuma, T Ohkusa, T Fujimori, Y Ono, M Pauly, C Faber, R Glaesener, A F P M de Goeij, J W Arends, S N Andersen, T Lövig, J Breivik, G Gaudernack, O P F Clausen, P De Angelis, G I Meling, T O Rognum, R Smith, H-S Goh, A Font, R Rosell, X F Sun, H Zhang, J Benhattar, L Losi, J Q Lee, S T Wang, P A Clarke, S Bell, P Quirke, V J Bubb, J Piris, N R Cruickshank, D Morton, J C Fox, F Al-Mulla, N Lees, C N Hall, D Snary, K Wilkinson, D Dillon, J Costa, V E Pricolo, S D Finkelstein, J S Thebo, A J Senagore, S A Halter, S Wadler, S Malik, K Krtolica, N Urosevic

**Affiliations:** Department of Medicine & Therapeutics, Imperial College School of Medicine, Chelsea & Westminster Hospital, 369 Fulham Road, London, SW10 9NH, UK; Royal Marsden Hospital, Sutton, UK; University of Western Australia, Nedlands, Australia; Bancroft Centre, Herston, Australia; Queensland University of Technology, Australia; Department of Medical Oncology, St Vincent's Hospital, Sydney, Australia; School of Pathology, University of New South Wales, Sydney, Australia; Charles University Hospital, Hradex Kralove, Czech Republic; Hospital Avicenne, Bobigny; Groupe Hospitalier Cochin-Saint Vincent de Paul, Paris, France; INSERM, Foundation J. Dausset, Paris, France; Max-Planck-Institut fur Molekulare Physiologie, Dortmund, Germany; Institute of Pathology, Ludwig-Maximilians-University of Munich, Germany; Department of Biological Chemistry, University of Athens, Athens, Greece; Department of Biology, Morrill Science Centre, University of Massachusetts, USA; Department of Pathology, Queen Mary Hospital, Hong Kong; Department of Surgery, Queen Mary Hospital, Hong Kong; Royal College of Surgeons, Dublin, Eire; St Vincent's Hospital, Dublin, Eire; Instituto Naziolale per la Ricerca sul Cancro, Genova, Italy; Department of Anatomy, surgery & Oncology, Palermo University, Italy; 1st Department of Surgery, School of Medicine, Kanazawa University, Japan; 2nd Department of Internal Medicine, Fukui Medical School, Japan; 1st Department of Internal Medicine, Tokyo Medical & Dental University, Japan; Department of Pathology, Dokkyo University School of Medicine, Tochigi, Japan; RCMS Lab, Fondation ‘Recherche Cancer et Sang’, Luxembourg; Department of Surgery, St-Theresia-Clinics, Luxembourg; Department of Surgery, the Hospital Centre of Luxembourg, Luxembourg; Department of Pathology, Masstricht University, The Netherlands; The National Hospital, Oslo, Norway; The Norwegian Radium Hospital, Oslo, Norway; Molecular Biology Laboratory, Tan Tock Seng Hospital, Singapore; Medical Oncology Service, Hospital Germans Trias I Pujol, Barcelona, Spain; Department of Oncology, University of Linkoping, Sweden; Department of Cell Biology, University of Linkoping; Institut Universitaire de Pathologie, Lausanne, Switzerland; Department of Surgery, National Cheng Kung University, Taiwan; Department of Public Health, National Cheng Kung University, Taiwan; Institute of Cancer Research, Sutton, UK; Molecular Oncology, University of Leeds, UK; Department of Pathology, University Medical School, Edinburgh, UK; Sir Alastair Currie CRC Laboratories, Edinburgh, UK; Queen Elizabeth Hospital, University of Birmingham, UK; Zeneca Diagnostics, Northwich, UK; Beatson Institute for Cancer Research, Glasgow, UK; Department of General Surgery, Wythenshawe Hospital, Manchester, UK; Imperial Cancer Research Fund, London; St Mark's Hospital, Harrow, UK; Yale University School of Medicine, CT, USA; Rhode Island Hospital, Brown University, USA; Department of Pathology, University of Pittsburgh Medical Centre; Ferbuson-Blodget Digestive Disease Institute, Grand Rapids, MI, USA; Vanderbilt University, Nashville, TN, USA; Albert Einstein College of Medicine, Bronx, NY, USA; Institute of Nuclear Sciences ‘Vinca’, Belgrade, Yugoslavia; Military Medical Academy, Belgrade, Yugoslavia

## Abstract

Researchers worldwide with information about the Kirsten ras (Ki-ras) tumour genotype and outcome of patients with colorectal cancer were invited to provide that data in a schematized format for inclusion in a collaborative database called RASCAL (The Kirsten ras in-colorectal-cancer collaborative group). Our results from 2721 such patients have been presented previously and for the first time in any common cancer, showed conclusively that different gene mutations have different impacts on outcome, even when the mutations occur at the same site on the genome. To explore the effect of Ki-ras mutations at different stages of colorectal cancer, more patients were recruited to the database, which was reanalysed when information on 4268 patients from 42 centres in 21 countries had been entered. After predetermined exclusion criteria were applied, data on 3439 patients were entered into a multivariate analysis. This found that of the 12 possible mutations on codons 12 and 13 of Kirsten ras, only one mutation on codon 12, glycine to valine, found in 8.6% of all patients, had a statistically significant impact on failure-free survival (*P* = 0.004, HR 1.3) and overall survival (*P* = 0.008, HR 1.29). This mutation appeared to have a greater impact on outcome in Dukes’ C cancers (failure-free survival, *P* = 0.008, HR 1.5; overall survival *P* = 0.02, HR 1.45) than in Dukes’ B tumours (failure-free survival, *P* = 0.46, HR 1.12; overall survival *P* = 0.36, HR 1.15). Ki-ras mutations may occur early in the development of pre-cancerous adenomas in the colon and rectum. However, this collaborative study suggests that not only is the presence of a codon 12 glycine to valine mutation important for cancer progression but also that it may predispose to more aggressive biological behaviour in patients with advanced colorectal cancer. © 2001 Cancer Research Campaign http://www.bjcancer.com


British Journal of Cancer (2001) 85(5), 692–696
© 2001 Cancer Research Campaign
doi: 10.1054/ bjoc.2001.1964, available online at http://www.idealibrary.com on http://www.bjcancer.com

Presented by the Kirsten ras in-colorectal-cancer collaborative group

### Kirsten ras mutations in patients with colorectal cancer: the 'RASCAL II' study

**HJN Andreyev[1,2], AR Norman[2], D Cunningham[2], J Oates[2], BR Dix[3], BJ Iacopetta[3], J Young[4], T Walsh[5], R Ward[6], N Hawkins[7], M Beranek[8], P Jandik[8], R Benamouzig[9], E Jullian[10], P Laurent-Puig[11], S Olschwang[11], O Muller[12], I Hoffmann[12], HM Rabes[13], C Zietz[13], C Troungos[14], C Valavanis[15], ST Yuen[16], JWC Ho[17], CT Croke[18], DP O'Donoghue[19], W Giaretti[20], A Rapallo[20], A Russo[21], V Bazan[21], M Tanaka[22], K Omura[22], T Azuma[23], T Ohkusa[24], T Fujimori[25], Y Ono[25], M Pauly[26], C Faber[27], R Glaesener[28], AFPM de Goeij[29], JW Arends[29], SN Andersen[30], T Lövig[30], J Breivik[31], G Gaudernack[31], OPF Clausen[30], P De Angelis[30], GI Meling[30], TO Rognum[30], R Smith[32], H-S Goh[32], A Font[33], R Rosell[33], XF Sun[34], H Zhang[35], J Benhattar[36], L Losi[36], JQ Lee[37], ST Wang[38], PA Clarke[39], S Bell[40], P Quirke[40], VJ Bubb[41], J Piris[42], NR Cruickshank[43], D Morton[43], JC Fox[44], F Al-Mulla[45], N Lees[46], CN Hall[46], D Snary[47], K Wilkinson[48], D Dillon[49], J Costa[49], VE Pricolo[50], SD Finkelstein[51], JS Thebo[52], AJ Senagore[52], SA Halter[53], S Wadler[54], S Malik[54], K Krtolica[55] and N Urosevic[56]**

[1]Department of Medicine & Therapeutics, Imperial College School of Medicine, Chelsea & Westminster Hospital, 369 Fulham Road, London SW10 9NH, UK, [2]Royal Marsden Hospital, Sutton, UK; [3]University of Western Australia, Nedlands, Australia; [4]Bancroft Centre, Herston, Australia; [5]Queensland University of Technology, Australia; [6]Department of Medical Oncology, St Vincent's Hospital, Sydney, Australia; [7]School of Pathology, University of New South Wales, Sydney, Australia; [8]Charles University Hospital, Hradex Kralove, Czech Republic; [9]Hospital Avicenne, Bobigny; [10]Groupe Hospitalier Cochin-Saint Vincent de Paul, Paris, France, [11]INSERM, Foundation J. Dausset, Paris, France; [12]Max-Planck-Institut fur Molekulare Physiologie, Dortmund, Germany; [13]Institute of Pathology, Ludwig-Maximilians-University of Munich, Germany; [14]Department of Biological Chemistry, University of Athens, Athens, Greece; [15]Department of Biology, Morrill Science Centre, University of Massachusetts, USA; [16]Department of Pathology, Queen Mary Hospital, Hong Kong; [17]Department of Surgery, Queen Mary Hospital, Hong Kong; [18]Royal College of Surgeons, Dublin, Eire; [19]St Vincent's Hospital, Dublin, Eire; [20]Instituto Naziolale per la Ricerca sul Cancro, Genova, Italy; [21]Department of Anatomy, surgery & Oncology, Palermo University, Italy; [22]1st Department of Surgery, School of Medicine, Kanazawa University, Japan; [23]2nd Department of Internal Medicine, Fukui Medical School, Japan; [24]1st Department of Internal Medicine, Tokyo Medical & Dental University, Japan [25]Department of Pathology, Dokkyo University School of Medicine, Tochigi, Japan; [26]RCMS Lab, Fondation 'Recherche Cancer et Sang', Luxembourg; [27]Department of Surgery, St-Theresia-Clinics, Luxembourg; [28]Department of Surgery, the Hospital Centre of Luxembourg, Luxembourg; [29]Department of Pathology, Masstricht University, The Netherlands; [30]The National Hospital, Oslo, Norway; [31]The Norwegian Radium Hospital, Oslo, Norway; [32]Molecular Biology Laboratory, Tan Tock Seng Hospital, Singapore; [33]Medical Oncology Service, Hospital Germans Trias I Pujol, Barcelona, Spain; [34]Department of Oncology, University of Linkoping, Sweden; [35]Department of Cell Biology, University of Linkoping; [36]Institut Universitaire de Pathologie, Lausanne, Switzerland; [37]Department of Surgery, National Cheng Kung University, Taiwan; [38]Department of Public Health, National Cheng Kung University, Taiwan; [39]Institute of Cancer Research, Sutton, UK; [40]Molecular Oncology, University of Leeds, UK; [41]Department of Pathology, University Medical School, Edinburgh, UK; [42]Sir Alastair Currie CRC Laboratories, Edinburgh, UK; [43]Queen Elizabeth Hospital, University of Birmingham, UK; [44]Zeneca Diagnostics, Northwich, UK; [45]Beatson Institute for Cancer Research, Glasgow, UK; [46]Department of General Surgery, Wythenshawe Hospital, Manchester, UK; [47]Imperial Cancer Research Fund, London; [48]St Mark's Hospital, Harrow, UK; [49]Yale University School of Medicine, CT, USA; [50]Rhode Island Hospital, Brown University, USA; [51]Department of Pathology, University of Pittsburgh Medical Centre; [52]Ferbuson-Blodget Digestive Disease Institute, Grand Rapids, MI, USA; [53]Vanderbilt University, Nashville, TN, USA; [54]Albert Einstein College of Medicine, Bronx, NY, USA; [55]Institute of Nuclear Sciences 'Vinca', Belgrade, Yugoslavia; [56]Military Medical Academy, Belgrade, Yugoslavia

**Summary** Researchers worldwide with information about the Kirsten ras (Ki-ras) tumour genotype and outcome of patients with colorectal cancer were invited to provide that data in a schematized format for inclusion in a collaborative database called RASCAL (The Kirsten ras in-colorectal-cancer collaborative group). Our results from 2721 such patients have been presented previously and for the first time in any common cancer, showed conclusively that different gene mutations have different impacts on outcome, even when the mutations occur at the same site on the genome. To explore the effect of Ki-ras mutations at different stages of colorectal cancer, more patients were recruited to the database, which was reanalysed when information on 4268 patients from 42 centres in 21 countries had been entered. After predetermined exclusion criteria were applied, data on 3439 patients were entered into a multivariate analysis. This found that of the 12 possible mutations on codons 12 and 13 of Kirsten ras, only one mutation on codon 12, glycine to valine, found in 8.6% of all patients, had a statistically significant impact on failure-free survival ($P$ = 0.004, HR 1.3) and overall survival ($P$ = 0.008, HR 1.29). This mutation appeared to have a greater impact on outcome in Dukes' C cancers (failure-free survival, $P$ = 0.008, HR 1.5; overall survival $P$ = 0.02, HR 1.45) than in Dukes' B tumours (failure-free survival, $P$ = 0.46, HR 1.12; overall survival $P$ = 0.36, HR 1.15). Ki-ras mutations may occur early in the development of pre-cancerous adenomas in the colon and rectum. However, this collaborative study suggests that not only is the presence of a codon 12 glycine to valine mutation important for cancer progression but also that it may predispose to more aggressive biological behaviour in patients with advanced colorectal cancer. © 2001 Cancer Research Campaign http://www.bjcancer.com

*Received 24 January 2001*
*Revised 14 May 2001*
*Accepted 15 May 2001*

*Correspondence to:* J Andreyev

It is widely accepted that mutations in the Kirsten ras (Ki-ras) gene in patients with colorectal cancer develop early in the progression from adenoma to carcinoma. Our first collaborative study including 2721 patients, clarified that Ki-ras mutations are not only

BJOC 01-1964 692-696 20/8/01 3:27 pm Page 693

important for the development of colorectal cancer but also for its progression (Andreyev et al, 1998). We showed that the presence of a mutation in Ki-ras increased risk of death by 26% ($P$ = 0.004). Secondly, for the first time in any common cancer, we conclusively showed that different mutations may have different effects. For example, any mutation of guanine (G) to thymidine (T) but not to adenine (A) or to cytosine (C) increased the risk of death by 44% ($P$ = 0.0002). When individual mutations were evaluated, one single mutation found in just under 10% of all patients with colorectal cancer – that of glycine to valine on codon 12 – was an independent risk factor for recurrence ($P$ = 0.0008) and death ($P$ = 0.0019). Thus, Ki-ras mutations were associated with an increased risk of relapse and death, and some mutations were found to be more aggressive than others.

The size of our collaborative database also allowed definitive conclusions to be drawn on a number of other unresolved issues. In particular, we found that mutations were not associated with gender, age, tumour site or Dukes' stage and that mutation rates seen in patients with sporadic tumours were comparable to the rates observed in patients with a predisposing cause for their cancer.

One intriguing possibility arising from our first study was that the aggressive mutations were playing a different role in early tumours compared to more advanced tumours. However, the first RASCAL (The Kirsten ras in-colorectal-cancer collaborative group) study was too small to define this point and it became obvious that additional patients would need to be recruited to the database so that this second study could explore further the role of the Ki-ras mutation at different stages of colorectal cancer.

### METHODS

### Patients

At least 2 invitations were sent to all researchers who had published original data in English or were known to have unpublished data on the significance of the Ki-ras gene in patients with colorectal adenocarcinoma. They were invited to participate in a collaborative register collecting original clinical data from such patients.

Participants were required to complete a questionnaire for each patient and details were entered into a database. All collaborators were asked to ensure that information in 3 areas in particular was as complete as possible. The following information was requested: (1) the genotype of the Ki-ras gene in the primary tumour at codons 12 and 13, (2) the date, Dukes' stage and apparent immediate outcome of any surgery for that cancer and (3) dates of follow-up and long-term outcome. Specific causes of death and dates of recurrence, if relevant, were also sought. All data were coded so that patient identity was only known to their physicians and were entered by one statistician (ARN) into a database called RASCAL.

### Statistics

Survival curves were generated using the product-limit method of Kaplan–Meier. The log rank test was used to evaluate differences in failure-free survival and overall survival curves. Failure-free survival was defined as the time from operation to relapse or death from any cause apart from peri-operative deaths. Overall survival was defined as the time from operation to death from any cause. Chi-square tests were used for comparison of categorical data. In view of the multiple statistical analyses performed and the large number of patients, only values where $P < 0.01$ were considered significant. Multivariate analysis was performed using Cox's model for proportional hazards survival analysis. Hazard ratios (HRs) and 95% confidence intervals (CIs) of the hazard ratios were calculated from the individual Cox multivariate analyses. All $P$-values were calculated from the improvement in log likelihood and were expressed as two-sided.

Bias introduced by the findings of different centres was examined using the test for heterogeneity across centres. The difference in prevalence of mutations between centres was examined using a chi-square test. Centre was treated as a random effect and age treated as a continuous variable in the model. Dukes' stage was analysed using dummy variables for each stage relative to Dukes' A. A separate model was generated for each of the mutation types. This allowed a hazard ratio to be calculated for each type separately, after controlling for centre, age and Dukes' stage. The model was repeated for the Dukes' B and then Duke's C patients separately to get an estimate of the effect on the individual stages.

### RESULTS

### Patient selection

Data on 4268 patients from 42 different centres in 21 countries were entered onto the RASCAL database (Table 1). Our earlier study had previously reported on 2721 of these in whom there had been clinical outcome data in 2445.

Patients excluded from further analysis were those with missing age ($n$ = 203) or Dukes' stage ($n$ = 75). Perioperative deaths ($n$ = 76) are all included in the database but the deaths are censored and do not count as events. Data from one centre ($n$ = 34) were removed as patient autopsy specimens were used. Centres that did not provide information about the exact mutation type were also excluded ($n$ = 488). Where a centre had a minority of missing mutation types, only the missing mutation patients were removed. Patients were also excluded if information provided for codon 12 included only mutations and data on codon 13 was missing ($n$ = 49 from 9 centres excluding 1.3–34.3% of their data). After these exclusions, the number of patients used for the analysis

**Table 1** Characteristics of 4268 patients from 42 different centres in 21 countries enrolled in the RASCAL database

| Characteristic | Male | Female | Unknown | Total |
|---|---|---|---|---|
| No. of patients | 2263 | 1977 | 28 | 4268 |
| Median age in years (range) | 68 (17–95) | 68 (19–103) | – | – |
| Dukes' stage | | | | |
| A | 334 | 304 | 5 | 643 |
| B | 941 | 819 | 9 | 1769 |
| C | 659 | 589 | 8 | 1256 |
| D | 287 | 238 | 0 | 525 |
| Unknown | 42 | 27 | 6 | 75 |
| Alive at last follow up | 1228 | 1137 | 26 | 2391 |
| Cause of death | | | | |
| Peri-operative | 48 | 28 | 0 | 76 |
| Cancer | 600 | 559 | 1 | 1160 |
| Unrelated to cancer | 191 | 116 | 0 | 307 |
| Unknown | 196 | 137 | 1 | 334 |

© 2001 Cancer Research Campaign 

**Table 2** Characteristics of 3439 patients from 35 centres in 19 countries included in the multivariate analysis

| Characteristic | Male | Female | Unknown | Total |
|---|---|---|---|---|
| No. of patients | 1824 | 1611 | 4 | 3439 |
| Median age in years (range) | 67 (17–95) | 69 (19–103) | – | – |
| Dukes' stage | | | | |
| A | 276 | 270 | 2 | 548 |
| B | 752 | 645 | 2 | 1399 |
| C | 558 | 498 | 0 | 1056 |
| D | 238 | 198 | 0 | 436 |
| Alive at last follow up | 984 | 923 | 4 | 1911 |
| Cause of death | | | | |
| Peri-operative | 44 | 22 | 0 | 66 |
| Cancer | 484 | 449 | 0 | 933 |
| Unrelated to cancer | 138 | 90 | 0 | 228 |
| Unknown | 174 | 127 | 0 | 301 |

presented here was 3439 from 35 centres in 19 countries (Table 2).

Bias introduced by the findings of different centres seemed to be of little significance as the test for heterogeneity across centres evaluated to $P = 0.22$, indicating that the data did not statistically differ between centres. However, when the prevalence of mutations between centres was examined, a value of $P < 0.001$ was obtained, suggesting a significant association between centre and mutation rate, perhaps because some centres used more sensitive techniques than others to detect mutations.

### Results of multivariate analysis

The age of the patient was a highly significant factor in our model. Even so, age barely influences the hazard ratios finally generated, as the estimations of mutation effects hardly differed whether age was or was not included.

We analysed the effects of a valine mutation in 2 different ways. First, we compared it to other mutations or wild type (no mutation) after controlling for Dukes' stage, age and centre (Table 3). A valine mutation reduced both failure-free ($P = 0.004$, HR 1.3, 95% CI 1.09–1.54) and overall survival ($P = 0.008$, HR 1.23, 95% CI 1.08–1.54). These data fall well within the CIs calculated from the smaller sample published in the 1st RASCAL study, where each centre was treated as a fixed effect rather than a random effect as used here. In addition, we again found that when guanine nucleotides were mutated to thymidine (but not adenine or cytosine) this also conveyed an adverse outcome on failure-free ($P = 0.002$, HR 1.27, 95% CI 1.1–1.47) and overall survival ($P = 0.002$, HR 1.28, 95% CI 1.1–1.5).

A second way to look at the same data was to compare the effect of all mutations together compared to wild type (Table 4). This puts all factors in at the same time and controls for them all. This model again found that only Dukes' stage, age and a valine mutation to be statistically significant independent risk factors for overall survival ($P = 0.008$, HR 1.29, 95% CI 1.08–1.55). The risk conveyed by the presence of a valine mutation in the data from individual centres is shown in Figure 1.

When the effects of a valine mutation on the Dukes' B (Table 5) and Dukes' C patients (Table 6) were analysed separately, we observed that the presence of a valine mutation in patients with Dukes' C carcinoma reduced the failure-free survival rate significantly ($P = 0.0076$, HR 1.5, 95% CI 1.13–1.98) and with a trend toward statistical significance when overall survival was considered ($P = 0.02$, HR 1.45, 95% CI 1.07–1.96). In contrast, the presence of a valine mutation had no effect on failure-free survival ($P = 0.46$, HR 1.12, 95% CI 0.84–1.46 and overall survival ($P = 0.36$, HR 1.15, 95% CI 0.86–1.53) in patients with Dukes' B carcinoma.

### DISCUSSION

This second RASCAL study is by far the largest study to date examining the impact of a mutation in the Ki-ras gene on the outcome of patients with colorectal cancer. Although two-thirds of

**Table 3** The 2nd RASCAL study: results of the 1st multivariate analysis including failure-free and overall survival

| | Total | Failure-free survival | | | | Overall survival | | | |
|---|---|---|---|---|---|---|---|---|---|
| Factor | *n* = | *n* = relapse or death | *P*-value | Hazard ratio | 95 % CI | *n* = deaths | *P*-value | Hazard ratio | 95 % CIs |
| Dukes' stage | | | | | | | | | |
| A | 548 | 148 | | 1 | | 129 | | 1 | |
| B | 1399 | 545 | < 0.0001 | 1.54 | 1.29–1.85 | 485 | < 0.0001 | 1.63 | 1.34–1.98 |
| C | 1056 | 610 | < 0.0001 | 3.42 | 2.85–4.09 | 540 | < 0.0001 | 3.37 | 2.78–4.09 |
| D | 436 | 291 | < 0.0001 | 9.74 | 7.95–11.93 | 274 | < 0.0001 | 11.65 | 9.40–14.44 |
| Age | 3439 | 1594 | < 0.0001 | 1.01 | 1.008–1.02 | 1428 | < 0.0001 | 1.02 | 1.01–1.02 |
| Hospital | 3439 | 1594 | < 0.0001 | 0.99 | 0.988–1.00 | 1428 | 0.0005 | 0.99 | 0.99–1.00 |
| Codon 12 mutations | | | | | | | | | |
| Valine | 300 | 148 | 0.004 | 1.30 | 1.09–1.54 | 129 | 0.008 | 1.29 | 1.08–1.55 |
| Aspartate | 354 | 171 | 0.613 | 1.04 | 0.89–1.22 | 143 | 0.441 | 0.94 | 0.79–1.11 |
| Cysteine | 92 | 54 | 0.447 | 1.11 | 0.85–1.46 | 51 | 0.169 | 1.26 | 0.93–1.62 |
| Serine | 75 | 42 | 0.035 | 1.42 | 1.04–1.93 | 34 | 0.300 | 1.20 | 0.86–1.70 |
| Alanine | 79 | 41 | 0.237 | 1.21 | 0.89–1.66 | 38 | 0.082 | 1.35 | 0.98–1.87 |
| Codon 13 mutations | | | | | | | | | |
| Aspartate | 297 | 149 | 0.469 | 0.94 | 0.79–1.12 | 139 | 0.437 | 0.93 | 0.78–1.12 |
| Mutation type | | | | | | | | | |
| Guanine to thymidine | 409 | 208 | 0.002 | 1.27 | 1.10–1.47 | 187 | 0.002 | 1.28 | 1.10–1.50 |
| Guanine to cytosine | 114 | 55 | 0.229 | 1.19 | 0.90–1.56 | 52 | 0.051 | 1.33 | 1.01–1.76 |
| Guanine to adenine | 713 | 355 | 0.381 | 1.06 | 0.94–1.19 | 309 | 0.626 | 0.97 | 0.85–1.10 |

This analysis compared the presence of a valine mutation to all other mutations or wild type after controlling for Dukes' stage, age and centre.

*© 2001 Cancer Research Campaign*

BJOC 01-1964 692-696 20/8/01 3:27 pm Page 695

**Table 4** 2nd RASCAL study: results of the 2nd multivariate analysis: overall survival. The effects of all mutations together compared to wild type. All factors are included simultaneously and the model controls for them all

| | **Total** | **Overall survival** | | | |
|---|---|---|---|---|---|
| **Factor** | ***n* =** | ***n* = deaths** | ***P-value*** | **Hazard ratio** | **95% CIs** |
| Dukes' stage | | | | | |
| A | 548 | 129 | – | 1 | – |
| B | 1399 | 485 | <0.0001 | 1.633 | 1.34–1.98 |
| C | 1056 | 540 | <0.0001 | 3.09 | 2.59–3.75 |
| D | 436 | 274 | <0.0001 | 11.65 | 9.4–14.44 |
| Age | 3439 | 1428 | <0.0001 | 1.018 | 1.01–1.02 |
| Hospital | 3439 | 1428 | 0.0005 | 0.993 | 0.99–1.00 |
| Codon 12 mutations | | | | | |
| Valine | 300 | 129 | 0.008 | 1.29 | 1.08–1.55 |
| Aspartate | 354 | 143 | 0.594 | – | – |
| Cysteine | 92 | 51 | 0.134 | – | – |
| Serine | 75 | 34 | 0.253 | – | – |
| Alanine | 79 | 38 | 0.064 | – | – |
| Codon 13 mutations | | | | | |
| Aspartate | 297 | 139 | 0.585 | 0.585 | – |

these patients were included in the first RASCAL study, this second study confirms that a glycine to valine mutation on codon 12 of the Ki-ras gene has a significant association with biological behaviour in colorectal cancer. The purpose of this second study was to explore the role of mutations at different stages of colorectal cancer and our findings suggest that this mutation is particularly aggressive in patients with Dukes' C cancer, in whom it is associated with a 50% increased risk of relapse or death.

We were unable to show a similar association between the presence of a valine mutation and outcome in patients with Dukes' B cancers. The explanation for this may be that there is no association between outcome and Ki-ras mutations in these patients. However, if abnormalities in the Ki-ras gene are important for the development of adenomas within the bowel, (Vogelstein et al, 1988), it is difficult to explain how they could be unimportant in less advanced cancers (Dukes' B) but still have a role in determining prognosis in more advanced cancers (Dukes' C). So, a second explanation for our lack of an association in Dukes' B tumours, is that despite the size of our colorectal cancer cohort, 4268 patients, our study was still significantly underpowered and a real difference has not been detected. Equally, our finding for the Dukes' C cancers may be a type 1 error, where a difference has been detected when none really exists.

When all valine mutations were examined together, our study approximately evaluates to 90% power to see a true effect. However, when the Dukes' C group tumours were considered, to

**Table 5** 2nd RASCAL study: results of the multivariate analysis when the effects of a valine mutation on the Dukes' B patients were analysed separately

| | **Total** | **Failure-free survival** | | | | **Overall survival** | | | |
|---|---|---|---|---|---|---|---|---|---|
| | ***n* =** | ***n* = relapse or death** | **P-value** | **Hazard ratio** | **95% CIs** | ***n* = deaths** | **P-value** | **Hazard ratio** | **95% CIs** |
| Dukes' stage B | 1399 | 545 | | | | 485 | | | |
| Codon 12 mutations | | | | | | | | | |
| Valine | 138 | 57 | 0.46 | 1.11 | 0.84–1.46 | 52 | 0.36 | 1.15 | 0.86–1.53 |
| Aspartate | 137 | 54 | 0.53 | 1.10 | 0.83–1.45 | 48 | 0.71 | 1.06 | 0.78–1.43 |
| Cysteine | 24 | 15 | 0.10 | 1.59 | 0.95–2.66 | 14 | 0.14 | 1.53 | 0.90–2.61 |
| Serine | 29 | 15 | 0.19 | 1.44 | 0.86–2.41 | 11 | 0.68 | 1.14 | 0.63–2.07 |
| Alanine | 28 | 10 | 0.55 | 1.22 | 0.65–2.28 | 8 | 0.66 | 1.18 | 0.58–2.37 |
| Codon 13 mutations | | | | | | | | | |
| Aspartate | 130 | 48 | 0.02 | 0.70 | 0.51–0.96 | 45 | 0.06 | 0.74 | 0.54–1.02 |
| Mutation type | | | | | | | | | |
| Guanine to thymidine | 168 | 74 | 0.08 | 1.25 | 0.98–1.59 | 69 | 0.11 | 1.24 | 0.96–1.60 |
| Guanine to cytosine | 44 | 16 | 0.30 | 1.30 | 0.80–2.12 | 15 | 0.25 | 1.37 | 0.82–2.30 |
| Guanine to adenine | 278 | 113 | 0.54 | 0.94 | 0.76–1.15 | 96 | 0.39 | 0.91 | 0.73–1.13 |

**Table 6** The 2nd RASCAL study: results of the multivariate analysis, when the effects of a valine mutation on Dukes' C patients were analysed separately

| | **Total** | **Failure-free survival** | | | | **Overall survival** | | | |
|---|---|---|---|---|---|---|---|---|---|
| | ***n* =** | ***n* = relapse or death** | **P-value** | **Hazard ratio** | **95% CIs** | **n = deaths** | **P-value** | **Hazard ratio** | **95% CIs** |
| Dukes' stage C | 1056 | 610 | 540 | | | | | | |
| Codon 12 mutations | | | | | | | | | |
| Valine | 84 | 54 | 0.008 | | 1.13–1.98 | 46 | 0.02 | 1.45 | 1.07–1.96 |
| Aspartate | 121 | 70 | 0.85 | 1.02 | 0.80–1.31 | 54 | 0.15 | 0.82 | 0.62–1.08 |
| Cysteine | 29 | 17 | 0.43 | 0.83 | 0.5 – 1.34 | 15 | 0.52 | 0.85 | 0.51–1.42 |
| Serine | 22 | 15 | 0.04 | 1.83 | 1.09–3.06 | 13 | 0.08 | 1.72 | 0.99–2.99 |
| Alanine | 20 | 12 | 0.82 | 1.07 | 0.60–1.90 | 11 | 0.72 | 1.12 | 0.61–2.03 |
| Codon 13 mutations | | | | | | | | | |
| Aspartate | 81 | 58 | 0.78 | 0.96 | 0.73–1.27 | 45 | 0.81 | 0.97 | 0.73–1.29 |
| Mutation type | | | | | | | | | |
| Guanine to thymidine | 119 | 70 | 0.10 | 1.24 | 0.97–1.58 | 63 | 0.13 | 1.23 | 0.95–1.60 |
| Guanine to cytosine | 28 | 16 | 0.79 | 1.07 | 0.66–1.73 | 16 | 0.62 | 1.14 | 0.69–1.87 |
| Guanine to adenine | 225 | 139 | 0.52 | 1.07 | 0.88–1.29 | 114 | 0.51 | 0.93 | 0.76–1.15 |

*© 2001 Cancer Research Campaign*

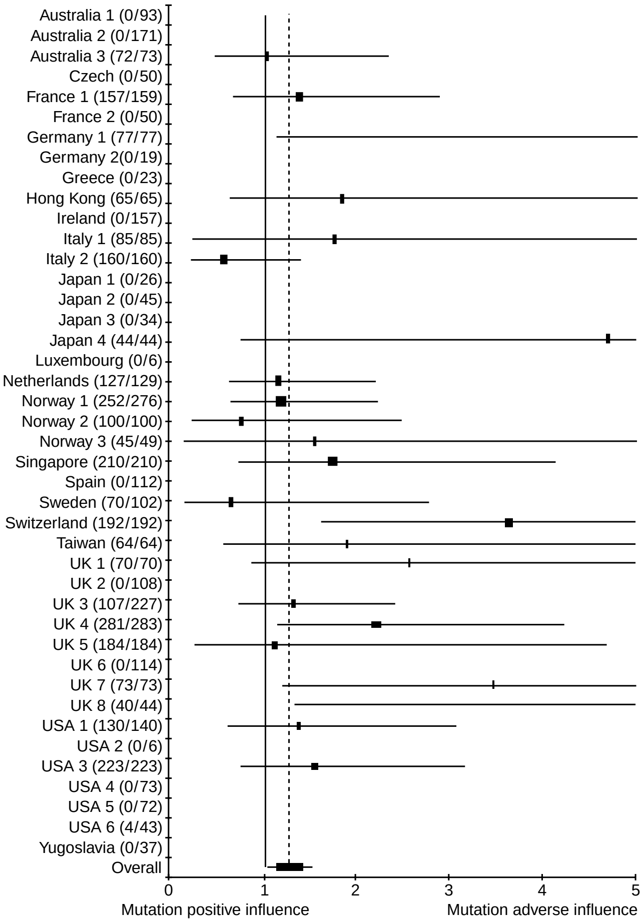

**Figure 1** Survival hazard ratios from data provided by the group collaborating in the RASCAL study, if a valine mutation is present. The box shows the relative size of the cohort from each centre; its position on the chart represents the degree of hazard conveyed by the presence of a valine mutation in the cohort from each centre providing survival data. The arms on either side of the box indicate the 95% CIs. Where no events occurred, or the centre reported no valine mutations, or the mutation types were not specified among the patients from a centre, no box is shown on this diagram. The number of patients included in the analysis/total number supplied by that centre is shown in brackets after the name of the centre detect a change in survival at 5 years from 40–50% with 90% power in patients with a valine mutation, compared to those with wild type or one of the other mutations, 1800 ‘events’ (relapses or deaths) would have had to occur in the Dukes’ C patients. We had 610 events. To detect a similar 10% improvement in 5-year survival in the Dukes’ B patients (from 60% to 70%) with 90% power, we needed 1045 events, while we had only 545.

Nevertheless, we have demonstrated that when researchers are willing to share original data to answer very specific issues – even data on individual patients – this gives studies great power that cannot be matched by individual groups. For example, the largest number of patients provided by any one author for this study was 283, so the only way to answer questions about the relationship between prognosis and individual mutations is through collaboration. The RASCAL studies also emphasize that it is no longer adequate to look for the presence or absence of mutations in tumours, but that instead research must concentrate on the individual mutations that are present. Up to 1 million people develop colorectal cancer annually worldwide. Of these, 86 000 have a valine mutation. This clinical study therefore, together with laboratory evidence, provides a real rationale for developing therapeutic strategies targeting this mutation.

Our findings from this clinical study support sound experimental evidence, reviewed in Al-Mulla et al (1999), that valine mutations produce proteins that behave differently to other mutated Ki-ras proteins. Different mutations within codon 12 lead to variation in the biological activity of mutated Ki-ras proteins by reducing GTPase activity and affinity for GTPase-activating proteins, preventing activation of GTPase-activating proteins and altering Ki-ras protein dissociation from binding proteins or downstream effectors. These critical molecular changes probably occur because of structural differences induced by mutations, particularly when the mutated Ki-ras protein is in its GTP-bound state.

In conclusion, this collaborative project has shown that a specific mutation in the Ki-ras gene at codon 12, found in 8.6% of all patients with colorectal cancer, increases the risks of recurrence or death by 30%. The presence of this mutation in Dukes’ C tumours is associated with a higher risk of recurrence or death, 50%.

### ACKNOWLEDGEMENTS

HJN Andreyev was funded by the British Digestive Foundation (now known as the Digestive Disorders Foundation).

### REFERENCES

Al-Mulla F, Milner-White EJ, Going JJ and Birnie GD (1999) Structural differences between valine12 and aspartate 12 Ras proteins may modify carcinoma aggression. *J Pathol* 1999; **187**: 4: 433–438

Andreyev HJN, Norman AR, Cunningham D et al (1998) Kirsten ras mutations in patients with colorectal cancer: the multicenter ‘RASCAL’ study. *J Natl Cancer Inst* 1998; **90 (9)**: 675–684

 *© 2001 Cancer Research Campaign*

